# Use of a Large Language Model as a Dermatology Case Narrator: Exploring the Dynamics of a Chatbot as an Educational Tool in Dermatology

**DOI:** 10.2196/72058

**Published:** 2025-09-16

**Authors:** Emmanouil Karampinis, Dafni Anastasia Bozi Tzetzi, Georgia Pappa, Dimitra Koumaki, Dimitrios Sgouros, Efstratios Vakirlis, Aikaterini Liakou, Markos Papakonstantis, Marios Papadakis, Dimitrios Mantzaris, Elizabeth Lazaridou, Enzo Errichetti, Cristian Navarrete-Dechent, Angeliki Victoria Roussaki Schulze, Alexandros Katoulis

**Affiliations:** 1 Second Dermatology Department, School of Health Sciences, Aristotle University of Thessaloniki, 54124, Thessaloniki, 84300, Greece, 30 6980420401; 2 Department of Dermatology, Faculty of Medicine, School of Health Sciences, University General Hospital of Larissa, University of Thessaly, Athens, Greece; 3 Department of Internal Medicine, General Hospital of Trikala, Trikala, Greece; 4 Second Department of Dermatology and Venereology, “Attikon” General University Hospital, Medical School, National and Kapodistrian University of Athens, Athens, 12462, Greece; 5 Dermatology Department, University Hospital of Heraklion, Heraklion, Greece; 6 First Department of Dermatology, Aristotle University Medical School, Thessaloniki, Greece; 7 First Department of Dermatology-Venereology, Faculty of Medicine, “Andreas Sygros” Hospital for Cutaneous and Venereal Diseases, National and Kapodistrian University of Athens, Athens, Greece; 8 Department of Dermatology, General Military Hospital of Athens, Athens, Greece; 9 Department of Surgery II, University of Witten Herdecke, Wuppertal, Germany; 10 Computational Intelligence and Health Informatics Lab, Nursing Department, University of Thessaly, Larissa, Greece; 11 Department of Experimental and Clinical Medicine, Institute of Dermatology, University of Udine, Udine, Italy; 12 Department of Dermatology, Escuela de Medicina, Pontificia Universidad Católica de Chile, Santiago, Chile

**Keywords:** chatbot, artificial intelligence, ChatGPT, dermatology, education, case reports, multiple choice answers, teaching methods

## Abstract

A comparison of dermatological cases generated by artificial intelligence (AI) versus those created without AI by medical students revealed that AI-created cases were characterized by detailed case descriptions, analysis of medical history, and clinical examinations, but lacked the depth, clinical relevance, and motivational elements found in non-AI cases, which were shorter, presented clinical dilemmas, and included challenging scenarios that students found more educational and engaging.

## Introduction

Artificial intelligence (AI) is no longer a futuristic concept but a present reality that has rapidly changed all aspects of life; health care is no exception. In medical education, AI offers tools that have the potential to outperform traditional methods of teaching and learning. Dermatology, a medical specialty that relies almost exclusively on visual recognition and clinical pattern analysis, provides fertile ground for AI to revolutionize how medical students and aspiring dermatologists are trained [1]. Chatbots, such as ChatGPT, can play a transformative role in the medical education of dermatology. They can serve as on-demand tutors, providing instant explanations for complex dermatological terms, clarifying concepts, or answering questions in real time. This capability allows medical students to explore topics and receive personalized support and guidance while studying. Along with supporting individual learning, the tools are also invaluable for educators, as they enable efficient creation of teaching materials. AI-driven models can generate quizzes, flashcards, summary notes, and even realistic and diverse clinical case scenarios [2,3]. The primary objective of this study was to assess whether large language models like ChatGPT-4 can create engaging, educational, and clinically relevant case-based scenarios for medical students.

## Methods

### Study Overview

Sixty-four medical students from two university-affiliated hospitals, Attikon University Hospital and University Hospital of Larissa, participated.

We assessed whether large language models like ChatGPT-4 can create engaging, educational, and clinically relevant case-based scenarios for medical students. “Engaging” refers to how much a case captures the learner’s interest, encourages active thinking, and motivates further exploration of the topic. Each AI-generated case was matched to a non-AI case on the same dermatological condition and similar educational objectives. Care was taken to ensure that both versions addressed comparable levels of difficulty, albeit with their inherent differences in style and formulation.

We developed a questionnaire featuring a mix of AI-generated and non-AI cases. The medical students were presented with multiple-choice questions, true/false statements, and correlation exercises. The AI-generated cases were created by instructing ChatGPT-4 to “generate an educational case in the form of multiple-choice questions for medical students” on the first attempt. Students graded each question on a Likert scale from 1 to 5, assessing how educational, motivational, or challenging they found the cases, without knowing the creator.

The final scores were evaluated regarding the normality with the Shapiro Wilk test, and then, the appropriate statistical tests were performed; for example, the Wilcoxon W test was the non-parametric test for paired data, as most comparisons were based on data with a significant departure from normality.

### Ethical Considerations

Participation was entirely voluntary and anonymous. All the participants provided consent, and the included data were de-identified. Institutional Review Board approval was not applicable, as it relied exclusively on voluntary and anonymous questionnaire responses, without the collection of identifiable or sensitive personal information.

## Results

Of 64 students, 45 answered the questionnaire (response rate of 70.3%). Among the 45 students, 36 (80%) reported using ChatGPT-4, 27 (60%) mentioned using Gemini-AI, while 9 (20%) students indicated they had never used a chatbot (the students could provide more than one answer). Twenty-five students (56%) stated that they used chatbots in their studies, though none reported using them in clinical practice.

Non-AI cases are thought to be more educational, motivational, and challenging in most scenarios, with statistical significance in many cases (Table 1). In contrast, in the case of True or False statements exercise and correlation test, no differences were detected between AI and non-AI examples. In the questionnaire students were asked about a conflicting situation involving multiple learning resources, including AI-based tools and traditional sources that provide different answers to the same question, 6.7% of students (3/45) would trust the AI answer, 68.9% of students (31/45) would trust the Internet source, and 24.4% of students (11/45) would further discuss the topic with a tutor.

**Table 1.** The median scores and range in different categories between artificial intelligence (AI) and non-AI cases on different skin diseases and lesions (Likert scale from 1 to 5).

	Educational	Motivational	Challenging
AI case on psoriasis	4 (4-5)	2 (2-5)	2 (1-4)
Non-AI case on psoriasis	4 (4-5)	5 (3-5)^a^	5 (3-5)^a^
AI case on atopic dermatitis	3 (3-4)	3 (3-4)	3 (2-3)
Non-AI case on atopic dermatitis	5 (4-5)^a^	5 (4-5)^a^	5 (4-5)^a^
AI case on rosacea	3 (3-4)	4 (3-4)	2 (2-3)
Non-AI case on rosacea	5 (4-5)^a^	5^a^	5 (4-5)^a^
AI case on HS^b^	3 (3-5)	4 (4-5)	4 (3-4)
Non-AI case on HS	4 (3-5)^a^	4 (3-5)	4 (4-5)
AI case on BCC^c^	3 (2-4)	3 (2-4)	3 (1-3)
Non-AI case on BCC	4 (3-4)	3 (2-5)	4 (3-4) ^a^
AI case on actinic keratosis	3 (1-3)	4 (3-5)	4 (2-4)
Non-AI case on actinic keratosis	5 (4-5)^a^	5 (4-5)^a^	5 (3-5)^a^
AI case on melanoma	4 (2-5)	3 (1-4)	2 (1-2)
Non-AI case on melanoma	5 (3-5)^a^	5^a^	5 (4-5)^a^
True or False statements question by AI	4 (2-4)	4 (2-4)	3 (1-5)
True or False statements question by clinician	4 (2-4)	4 (2-5)	4 (2-5)
Correlation question by AI	4 (3-5)	4 (3-4)	2 (1-3)
Correlation question by clinician	4 (3-5)	4 (3-4)	3 (2-4)

a Statistical significant in favor of non-AI example(*P*<.05)

b HS: hidradenitis suppurativa.

c BCC: basal cell carcinoma.

## Discussion

In dermatology, no studies have directly compared AI-generated case scenarios with those authored by clinicians in terms of the educational value, clinical relevance, or student engagement. In our study, AI-created cases were characterized by detailed case descriptions, analysis of medical history, clinical examinations, and follow-up questions but lacked the depth, clinical relevance, and motivational elements found in non-AI cases. Non-AI cases were shorter, presented clinical dilemmas, offered direct questions, and included challenging scenarios that students found more educational and engaging [4,5]. Non-AI questions, such as those comparing sebaceous hyperkeratosis and melanoma, differentiating between atopic dermatitis and scabies in a child patient, searching for comorbidities in rosacea, and critically evaluating a patient with hidradenitis suppurativa, were assessed with higher scores compared to the straightforward cases by AI chatbots. However, this observation does not imply that AI-generated questions have no value, as they can help students test their understanding. Tutors are also encouraged to make their own questions that simulate real-life scenarios that challenges students and do not rely blindly on chatbots for the quick production of exercises.

## Abbreviations

AI: artificial intelligence
